# Functional analyses of bacterial NanoRNase B proteins reveals defining features of this enzyme family

**DOI:** 10.1093/nar/gkaf1384

**Published:** 2025-12-17

**Authors:** Tanner M Myers, Andrew A Burnim, Madison D Jermain, Holger Sondermann, Vincent T Lee, Xiaofang Jiang, Wade C Winkler

**Affiliations:** Department of Chemistry and Biochemistry, University of Maryland, College Park, Maryland 20742, United States; Intramural Research Program, National Library of Medicine, National Institutes of Health, Bethesda 20894, MD, United States; Department of Cell Biology and Molecular Genetics, University of Maryland, College Park, Maryland 20742, United States; CSSB—Centre for Structural Systems Biology, Deutsches Elektronen-Synchrotron (DESY), Hamburg 22607, Germany; Christian-Albrechts-Universität, Kiel 24118, Germany; Department of Cell Biology and Molecular Genetics, University of Maryland, College Park, Maryland 20742, United States; Intramural Research Program, National Library of Medicine, National Institutes of Health, Bethesda 20894, MD, United States; Department of Chemistry and Biochemistry, University of Maryland, College Park, Maryland 20742, United States; Department of Cell Biology and Molecular Genetics, University of Maryland, College Park, Maryland 20742, United States

## Abstract

A combination of exoribonucleases and endoribonucleases degrades RNA polymers to recycle nucleoside monophosphates. A byproduct of these reactions is the accumulation of short RNAs, 2–5 nucleotides in length. Characteristic enzymes, generally referred to as nanoRNases, specifically process short RNAs. Genes encoding nanoRNases are essential in some bacteria; therefore, it is assumed that the accumulation of short RNAs is detrimental to cells. However, the substrate preferences and enzymatic mechanisms of the known categories of nanoRNase enzymes have not been equally investigated. The NrnB category of nanoRNases has been particularly understudied. In this study, we identified bacterial NrnB homologs and discovered they can be grouped into three classes of proteins, which can be identified by their characteristic sequence features. Purified representatives of these classes of proteins revealed that they all process RNA substrates from the 3′-terminus. The presence of sequence features at the C-terminus was shown to be diagnostic for general exoribonuclease activity against long RNA substrates, whereas the absence of these C-terminal elements was correlated with proteins that preferentially acted against shorter RNA substrates. Together, these data define members of the overall NrnB family of nanoRNase proteins and identify some of their key features.

## Graphical Abstract

**Graphical Abstract:**
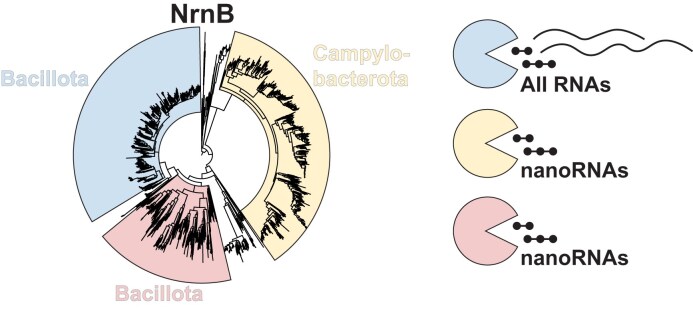

## Introduction

RNA degradation is a fundamental process for all organisms. A combination of endo- and exoribonucleases systematically degrades RNA molecules, resulting in the release of monoribonucleotides and short RNA fragments 2–5 nucleotides in length. NanoRNases are a specialized subclass of broadly conserved exoribonucleases that degrade short oligoribonucleotide products that arise from the breakdown of long RNA transcripts and diribonucleotides produced by the linearization of cyclic dinucleoside monophosphate second messenger signaling molecules (i.e. c-di-GMP, c-di-AMP, and cGAMP) [1]. Accumulation of short RNAs alters cellular physiology in multiple ways, including biofilm production, cellular motility, sporulation efficiency, growth rate, and virulence [2–13]. The genes encoding general 3′-5′ exoribonucleases are often dispensable [14–16]. For example, deletion of multiple exoribonuclease genes from *Escherichia coli* or *Bacillus subtilis* does not result in a significant defect in growth rate [17, 18]. In contrast, nanoRNase-encoding genes are oftentimes essential for cell growth, although the basis for this essentiality has not yet been determined [8, 10, 19, 20]. For all these reasons, it can be concluded that the degradation of short RNA oligonucleotides represents a discrete yet pivotal step in RNA metabolism.

The enzymes known to function in the clearance of short RNAs are Oligoribonuclease (Orn), NanoRNase A (NrnA), NanoRNase B (NrnB), and NanoRNase C (NrnC). Orn and NrnC proteins possess similar DEDD active-site motifs and share structural features, as they belong in the DnaQ-like superfamily of proteins, the family of proteins that is best represented by the epsilon 3′-5′ exonuclease subunit of DNA polymerase. Recent structural analyses of Orn and NrnC demonstrated that these enzymes exhibit a preference for diribonucleotides over other short RNAs owing to active site features that restrict the binding of longer RNAs [20, 21]. The preference for dinucleotides was further supported by biochemical assays, which showed that Orn and NrnC rapidly process dinucleotides at a much faster rate than they do longer RNA substrates. However, these observations do not necessarily indicate that all nanoRNases specifically process dinucleotide substrates; indeed, the substrate preferences of NrnA and NrnB proteins are not as well understood.

Historically, NrnA, NrnB, and NrnC have been less studied than the Orn proteins. NrnA and NrnB are members of the same protein family and contain the DHH domain [22]. The superfamily of phosphoesterase proteins that contains the DHH domain (InterPro entry IPR001667) includes but is not limited to the bacterial DNase RecJ and inorganic pyrophosphatase enzymes. However, many DHH proteins also feature a second domain referred to as the DHH-associated domain (DHHA1; InterPro entry IPR003156). Some of these DHH–DHHA1 proteins include additional domains, such as the PAS and GGDEF domains found in cyclic di-adenosine monophosphate-cleaving GdpP proteins [23]. There are yet other architectures associated with DHH–DHHA1 domains, such as those found in alanyl-tRNA synthetases or tRNA nucleotidyltransferases. However, many DHH–DHHA1 proteins lack additional domains and can therefore be referred to as “standalone” DHH–DHHA1 proteins. The NrnA and NrnB nanoRNases are widespread examples of standalone DHH–DHHA1 proteins [12, 13].

*Bacillus subtilis* NrnA was previously shown to preferentially process linear nucleic acid substrates between 2–4 nucleotides in length [13]. Yet not all NrnA homologs behave identically. For example, some Actinobacteria encode NrnA paralogs named CnpB, which specialize in the processing of cyclic di-adenosine monophosphate (c-di-AMP) to AMP [24, 25]. It can be assumed that other classes of NrnA-specialized paralogs still await discovery, such as the recent demonstration that *Vibrio cholerae* encodes an NrnA-like protein that specifically processes the linear diribonucleotide pGpG [26]. For all these reasons, annotation of NrnA proteins remains highly incomplete. Annotation of NrnB proteins is even more problematic. Little data have been published on the phylogenetic distribution of NrnB homologs. In addition, only a few publications have described any biochemical characterization of representative proteins. For these reasons, there is a large gap in our knowledge regarding the distribution, relative importance, and biochemical activities of standalone DHH–DHHA1 proteins corresponding to NrnB.

It has been speculated that NrnA and NrnB may be redundant in their enzymatic activities and intracellular functions [27, 28]. In a prior study, we purified NrnA and NrnB proteins from *B. subtilis* and subjected them to biochemical and biophysical assays. These data demonstrated that *B. subtilis* NrnA (NrnA*_Bs_*) specifically degraded RNAs 2–4 nucleotides in length using a 5′-3′ exonucleolytic mechanism and is expressed during vegetative growth [13]. In contrast, *B. subtilis* NrnB (NrnB*_Bs_*) showed significant and surprising differences compared with NrnA*_Bs_*. NrnB*_Bs_* is specifically expressed during sporulation and degrades RNAs of varying lengths, from very short to long, utilizing a 3′-5′ exonucleolytic mechanism [12]. Structural data on NrnB proteins are lacking; therefore, it is unknown what features are responsible for differentiating *B. subtilis* NrnA and NrnB proteins such that the latter possesses the ability to process both short and long RNA substrates. Nor is it clear what structural features participate in orienting the proteins to either the 5′ (e.g. NrnA*_Bs_*) or 3′ (e.g. NrnB*_Bs_*) terminus for the processing of RNA substrates.

While these data helped identify the proteins that contribute to the degradation of short RNAs in *B. subtilis*, it was less clear how generally relevant these findings would be for other NrnB homologs. For instance, the biochemical differences between *B. subtilis* NrnA and NrnB proteins have raised several questions. Is the *B. subtilis* NrnB protein alone in its ability to recognize both short and long substrates, or is that a universal feature of NrnB homologs? Is the processing of RNA substrates from the 3′ terminus a general feature of NrnB proteins, or a feature of a unique subset of NrnB proteins that happens to include the *B. subtilis* variant? And, finally, can sequence features be identified that are diagnostic for NrnB functions? Answers to these questions would greatly improve the annotation of bacterial NrnA and NrnB proteins and enhance our understanding of the biological role(s) of nanoRNases. In this study, we used bioinformatic approaches to identify NrnB homologs and examine their phylogenetic distribution. We then purified diverse NrnB homologs for biochemical analyses to identify the enzymological features that define NrnB proteins.

## Materials and methods

### Bacterial strains and culture conditions

*Escherichia coli* strains were grown in LB containing (as needed) 100 μg/ml carbenicillin at 37°C in a shaking incubator. *Bacillus subtilis* strains were grown in LB in the presence of 5 μg/ml chloramphenicol, or 100 μg/ml spectinomycin. The method utilized to make deletion strains of *B. subtilis* Δ*nrnA, ΔnrnB*, and Δ*nrnA*Δ*nrnB* is described previously [11]. To build *E. coli* overexpression strains for protein purification, the genes encoding *nrnB_Bl_, nrnB_Bt_*, and *nrnB_Hp_* were purchased from Integrated DNA Technologies (IDT) as codon-optimized gene fragments and were subcloned into their respective expression vector by Gibson assembly [29]. These genes encoded *nrnB* homologs from *Bacillus licheniformis, Bacillus thuringiensis*, and *Helicobacter pylori*, respectively. The gene encoding *nrnB_Bl_* was cloned in-frame into an expression vector so that this protein would be expressed without any affinity tags. The gene encoding *nrnB_Bt_* was cloned in-frame into the plasmid pHisll to be expressed with a C-terminal 6× His tag. The gene for *nrnB_Hp_* was cloned in-frame to be expressed with a cleavable N-terminal 10× His-SUMO tag. Complementation plasmids contained inducible copies of *nrnB_Bm_* (*Bacillus megaterium* NrnB), *nrnB_Hp_*, or *nrnB_Cj_* (*Campylobacter jejuni* NrnB), which were purchased from IDT as codon-optimized gene fragments. The sequences for *nrnB_Bm_, nrnB_Bm_*_ΔC-term_ (residues 1–306), *nrnB_Bt_, nrnB_Hp_*, or *nrnB_Cj_* were then subcloned via Gibson assembly into the plasmid pDR111, which harbors an isopropyl β-D-1-thiogalactopyranoside (IPTG)-controllable promoter region upstream of the target gene, flanked by regions of homology to the nonessential *amyE* locus for ectopic integration. All transformations of *B. subtilis* were performed using a previously described protocol [30]. *Escherichia coli* XL10-Gold (Agilent) was initially transformed with all plasmids, and the sequences of all inserts were verified by Sanger sequencing. *Escherichia coli* T7 Express (NEB) was transformed with all plasmids that were used for overexpression and purification of target proteins.

### Protein overproduction and purification

*Escherichia coli* strains harboring the different overproduction expression vectors were cultured, shaking, overnight at 37°C. The next day, the cultures were diluted into fresh 2x yeast extract tryptone medium (2xYT) containing 100 µg/ml carbenicillin and were grown with shaking until an OD_600_ of ~0.4–0.8 was reached. At this point, cells harboring plasmids to produce either tagless-NrnB*_Bl_*, 10× His-SUMO–NrnB*_Hp_*, or C-terminal 6× His–NrnB*_Bt_* were removed from the 37°C incubator, induced with 1 mM IPTG, and grown shaking at room temperature overnight for ~16–18 h. Cells were harvested by centrifugation and resuspended at a ratio of 1 g cell pellet to 10 ml of lysis buffer. The cells overexpressing NrnB*_Bl_*, NrnB*_Bt_*, or 10× His-SUMO–NrnB*_Hp_* were resuspended in 25 mM Tris–HCl (pH 8.0), 300 mM NaCl, and 5% glycerol (v/v). Cells overexpressing the different proteins were lysed by sonication, and the lysates were clarified by two rounds of centrifugation at 12 000 rpm for 15 min. At this point, polyethyleneimine was added to a final concentration of 0.5% (w/v) to the C-terminal 6× His–NrnB*_Bt_* preparation. C-terminal 6× His–NrnB*_Bt_* was centrifuged at 10 000 rpm for 10 min to remove the polyethyleneimine. Next, solid ammonium sulfate was added to reach 70% saturation. The 6× His–NrnB*_Bt_* precipitated and was resuspended in 25 mM Tris–HCl (pH 8.0), 300 mM NaCl, 10 mM imidazole, and 5% glycerol. The 10× His-SUMO–NrnB*_Hp_* or C-terminal 6× His–NrnB*_Bt_* clarified lysates were incubated with cOmplete™ His-Tag Purification Resin for 1 h rocking on ice. After incubation, the resin was washed with 10 column volumes of wash buffer 1 (25 mM tris–HCl, pH 8.0, 300 mM NaCl, 10 mM imidazole, and 5% glycerol). Next, the column was washed with five column volumes of wash buffer 2 (25 mM tris–HCl, pH 8.0, 300 mM NaCl, 25 mM imidazole, and 5% glycerol). Finally, 10× His-SUMO–NrnB*_Hp_* or NrnB*_Bt_* was eluted with five column volumes of elution buffer (25 mM tris–HCl, pH 8.0, 300 mM NaCl, 250 mM imidazole, and 5% glycerol). Following the nickel column, purified 10× His-SUMO–NrnB*_Hp_* or NrnB*_Bt_* was dialyzed into 25 mM tris–HCl (pH 8.0), 300 mM NaCl, and 5% glycerol. At this point, NrnB*_Bt_* was aliquoted and flash-frozen in liquid nitrogen. 10× His-SUMO–NrnB*_Hp_* was subjected to tag removal overnight on ice by the addition of 10× His–bdSENP1 in the presence of 2 mM MgCl_2_ and 2 mM dithiothreitol (DTT) [31]. Following tag removal, NrnB*_Hp_* was incubated with cOmplete™ His-Tag Purification Resin for 45 min to remove the 10× His–bdSENP1 and the 10× His-SUMO tag. Untagged NrnB*_Hp_* was eluted in the flow-through, dialyzed into 25 mM tris–HCl (pH 8.0), 300 mM NaCl, and 5% glycerol, and aliquoted and flash-frozen in liquid nitrogen. All protein samples were stored at −80°C. Tagless-NrnB*_Bl_* was purified using a previously published protocol for purification of NrnB*_Bs_* [12].

### Preparation of whole cell lysates

Overnight cultures of *B. subtilis* strains were diluted in fresh LB to an OD_600_ of 0.1 and incubated, shaking at 37°C. Once the cells reached an OD_600_ of ~1.0, 250 µM IPTG was added to the cultures and the cells were cultured for an additional 40 min. Cells were collected by centrifugation and resuspended to concentrate the sample 10-fold in 25 mM tris–HCl (pH 8.0), 100 mM NaCl, and 0.1 mM phenylmethanesulfonyl fluoride (PMSF). The cells were lysed by sonication, and the resulting cellular lysates were individually aliquoted and stored at −80°C.

### Oligoribonucleotide labeling

Chemically synthesized RNAs (2–7, 20, and 44-mers) were subjected to 5′ labeling with T4 Polynucleotide Kinase (PNK, New England Biolabs). Each RNA sample was incubated at an equimolar concentration with either [γ-^32^P]-ATP or ATP. Reactions for radiolabeling RNAs were conducted at a final concentration of 0.5 µM, while the reactions for phosphorylating cold RNAs were at a final concentration of 2 µM. These reactions proceeded by incubation at 37°C for 1 h prior to heat inactivation of T4 PNK by incubation at 65°C for 20 min. The 5′-^32^P 44-mer was labeled with T4 PNK and residual ATP was removed using a Zymo-RNA clean and concentrator kit. Because of the length of the shorter RNAs, we observed <100% labeling, and residual [γ-^32^P]-ATP was observed at the solvent front of the denaturing polyacrylamide gels.

### RNase activity assays

Trace amounts of 5′-^32^P-RNAs 2–7, 10, and 20 nucleotides in length (40 nM of each RNA) were simultaneously subjected to cleavage by 50 nM of the respective protein in reactions containing 25 mM tris–HCl (pH 8.0), 100 mM NaCl, 5 mM MgCl_2_ in Fig. 3A or in the presence of 5 mM MnCl_2_ in Fig. 3B. For reactions in Fig. 3C, 1 µM of 5′-phosphorylated unstructured 44-mer with trace amounts (40 nM of 5′-^32^P-44-mer) was incubated with 100 nM of the indicated protein in a reaction containing 25 mM tris–HCl, 100 mM NaCl, 5 mM MgCl_2_. In Fig. 4A, 400 nM of protein was incubated with 40 nM of 5′-^32^P-20-mer harboring an internal phosphorothioate linkage in 25 mM tris–HCl (pH 8.0), 100 mM NaCl, and 5 mM MnCl_2_. For the cellular lysate-based RNA reactions in Fig. 6, trace amounts (40 nM) of 5′-^32^P-20-mer or the dinucleotide 5′-^32^P-pGpG was simultaneously incubated with whole cell lysates. These reactions also consisted of 25 mM tris–HCl (pH 8.0), 100 mM NaCl, 10 mM MgCl_2_, and 50 µM MnCl_2_. Reactions in Figs 7 and 8 were conducted in the presence of 25 mM tris–HCl (pH 8.0), 100 mM NaCl, 5 mM MnCl_2_. All cleavage reactions were quenched in 4 M urea with 150 mM ethylenediaminetetraacetic acid (EDTA), and degradation products were resolved via denaturing 20% polyacrylamide gel electrophoresis (PAGE). Gels were imaged using a Cytiva Amersham Typhoon™ laser scanner platform. Gel quantification was conducted using FIJI software [32].

**Figure 1.**
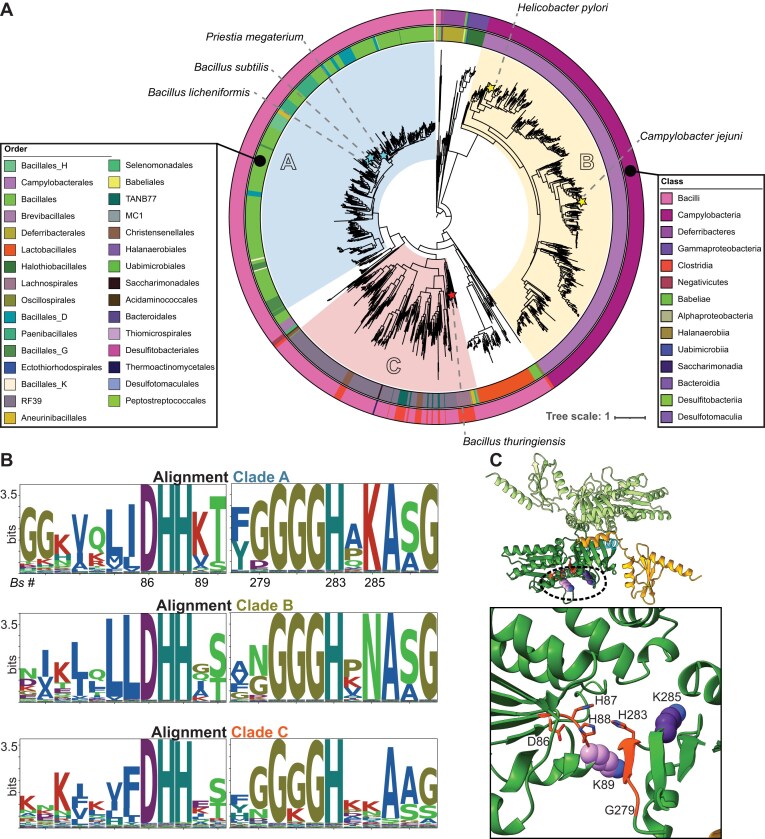
Phylogenetic and sequence analysis of NrnB sequences. (**A**) Maximum likelihood phylogenetic tree of NrnB sequences rooted at the midpoint. Three major clades are shown and colored by blue, yellow, and red, respectively. The ancestor nodes of interest for each major clade are shown. Stars at the sequence tips indicate proteins that were investigated in this analysis. The outer rings correspond to the taxonomic class and order, respectively, of the host organism of the sequence. As indicated in the key, colors correspond to different Order and Class categories. (**B**) Active site conservation among NrnB sequences. Protein sequences corresponding to the three clades were used to construct multiple sequence alignments, which were then used to acquire information about putatively conserved residues. Shown in this figure are the conservation patterns for active site residues of the DHH and DHHA1 portions of NrnB proteins. The top sequence logo shows the corresponding numbering of residues for *B. subtilis* NrnB. (**C**) As described in the text and methods, AlphaFold3 was used to estimate the structural configuration of a dimeric NrnB*_Bs_* assembly. The inset shows a close-up of the putative active site residues.

### C-di-AMP cleavage assay

NrnB-like proteins were analyzed indirectly for c-di-AMP processing based on a coupled enzyme reaction where c-di-AMP linearization results in exposed 5′ phosphate, which is readily liberated by alkaline phosphatase. The release of inorganic phosphate was quantified using the Sigma–Aldrich^®^ Malachite Green Phosphate Assay Kit (MAK307). Five hundred nanomolar of the respective protein was incubated with 20 µM of c-di-AMP in the presence of 25 mM tris–HCl (pH 8.0), 100 mM NaCl, 5 mM MgCl_2_, and 10 U of alkaline phosphatase in a 40 µl reaction volume. After a 30-min incubation, reactions were stopped by the addition of 10 µl of quenching solution. Quenched samples were left for 30 min prior to reading the absorbance at 620 nm. The amount of phosphate release was correlated to a standard curve of inorganic phosphate.

### 2-Aminopurine release assay

Cellular lysates were incubated with 5 µM of the dinucleotide p(2AP)pG in the presence of 25 mM tris–HCl (pH 8.0), 100 mM NaCl, 5 mM MgCl_2_ in a black-walled 384-well plate. Reactions proceeded in a Spectramax M5 plate reader using the fluorescence excitation at 310 nm and the emission wavelength at 375 nm. We note that it was difficult to obtain a true *T* = 0 time point due to the lag between loading the sample and the initial readings on the plate reader.

### Maximum likelihood phylogenetic analysis of NrnB sequences

Protein sequences were systematically retrieved from two specialized databases to ensure a comprehensive analysis of both prokaryotic and eukaryotic proteins. From the Genome Taxonomy Database (GTDB) (release r214), prokaryotic protein sequences were selected based on their match to the SSF64182 (DHH phosphoesterase superfamily) profile based on InterProScan (version 5.60-92.0) and their classification within the COG2404 group as determined by eggNOGmapper (version 2.1.3) [33–35]. Similarly, eukaryotic protein sequences from the EukProt database were identified by their alignment with the SSF64182 profile via InterProScan [36]. All selected protein sequences needed to cover the “DHH” and “GGGH” domain signatures defined by the SSF64182 hidden Markov model.

To reduce sequence redundancy and improve the computational efficiency of subsequent analyses, protein sequences from the GTDB database were clustered at a 70% identity threshold using CD-HIT (version 4.8.1), whereas sequences from EukProt were clustered at a 90% identity threshold [37]. After clustering, the curated sequences from both databases were merged and aligned using MUSCLE (version 5.1) to ensure accurate homology representation [38]. Sites within the alignment exhibiting >95% gaps were removed to enhance the quality of the phylogenetic analysis. The final phylogenetic tree was constructed using FastTree (version 2.1.11) with default parameters, and the results were depicted in Supplementary Fig. S1 using iTOL [39].

**Figure 2.**
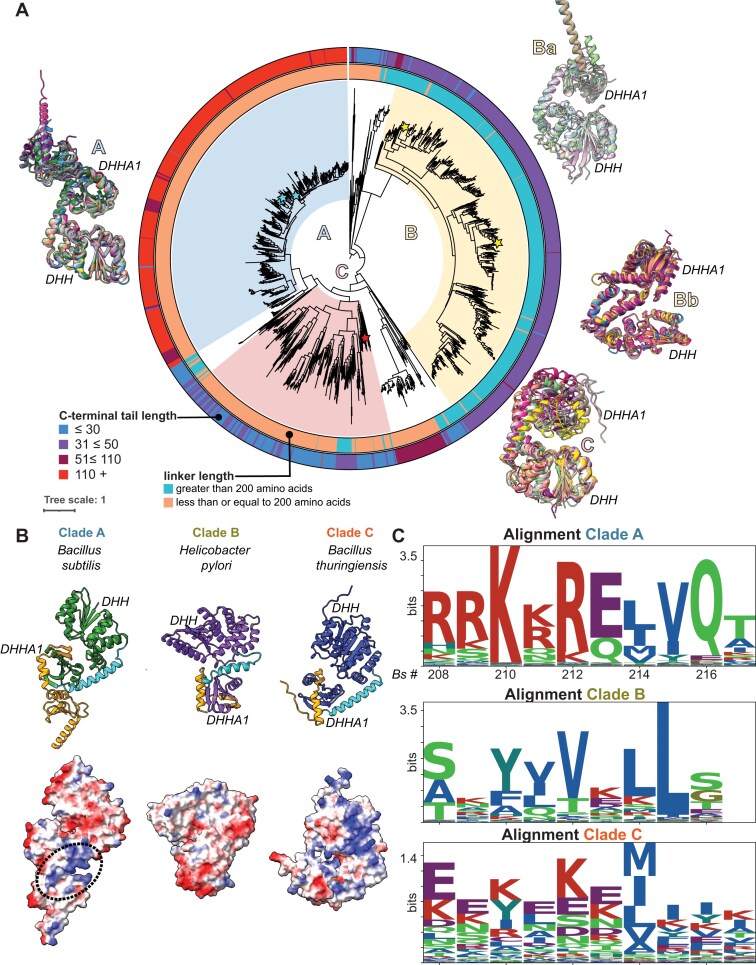
Clade-specific structural and sequence features across NrnB. (**A**) Phylogenetic tree highlighting structural variations within NrnB proteins. The outer ring represents the length of the C-terminal tail, while the inner ring categorizes the linker lengths connecting the DHH and DHHA1 domains into two groups: those longer than 200 amino acids (blue) and those 200 amino acids or shorter (peach). As discussed in the text, a sampling of ~15 protein sequences was randomly selected (Supplementary Fig. S2) from each clade, analyzed by AlphaFold3, overlayed if possible, and then displayed adjacent to the phylogenetic figure. (**B**) Representative AlphaFold3 models of NrnB monomers from *B. subtilis* (Clade A), *H. pylori* (Clade B), and *B. thuringiensis* (Clade C). Corresponding electrostatic potential maps below each model illustrate the charged surfaces, with the region of interest in *B. subtilis* NrnB highlighted by a dotted line. (**C**) Sequence logos display the alignment entropy for each clade. Alignment entropy is a measure of uncertainty or variability; therefore, these data show measurements of conservation and variability of key residues within the clades.

To further characterize the specific clade of interest, which included the *B. subtilis* NrnB sequence, we refined our analysis approach based on the initial phylogenetic tree constructed from sequences clustered at a 70% identity threshold. For this focused study, we retrieved all sequences represented by the initial tree’s nodes within this clade for a detailed examination. Multiple sequence alignments were then performed using ClustalO (version 1.2.4) [40]. Subsequently, a more refined phylogenetic tree was constructed using IQ-TREE (version 2.3.5), employing the model selection option “-m TEST” and bootstrap analysis with 1000 replicates (“-B 1000”) [41]. Additionally, we mapped features, including the length of the C-terminus and the linker regions, along with taxonomic information, onto the tree using iTOL (Figs 1A and 2A).

### Sequence alignment generation for NrnB clades

Following the initial alignment with ClustalO, the alignment for sequences from each NrnB clade—NrnB-A, NrnB-B, and NrnB-C—were further analyzed using Goalign (version 0.3.7) [42]. For each clade, sequence logos were generated using LogoMaker to visually represent the conservation and variability of amino acid residues across the sequences [43]. The entropy values were calculated for each position in the alignment to quantitatively assess the conservation level within the clades.

## Results

### Maximum likelihood phylogenetic tree of bacterial NrnB sequences

There is a significant gap in our knowledge regarding the distribution, relative importance, and biochemical activities of NrnB proteins. To address this issue, we sought to identify NrnB sequences from both prokaryotic and eukaryotic protein sequence databases by selecting those that belong to the DHH phosphoesterase superfamily and are categorized under COG2404. We refined the sequences to include only those containing both the DHH domain and GGGH motif. This analysis revealed that NrnB homologs can be found in all three domains of life, although a significant majority of the sequences corresponded to archaea or bacteria (Supplementary Fig. S1). In particular, we observed a major branch that includes the *B. subtilis* NrnB sequence. We retrieved all protein sequences from representative genomes for this branch, constructed a new phylogenetic tree, and performed a focused analysis (Fig. 1A), which revealed three major clades (Fig. 1A). Two of these clades were represented by organisms from the phylum Bacillota (labeled “A” and “C” in Fig. 1A), whereas the third clade is restricted to the Campylobacterota phyla (labeled “B” in Fig. 1A). To ascertain whether our prior biochemical observations on *B. subtilis* NrnB [12] are broadly representative of bacterial NrnB proteins, we focused our current investigation on these three clades.

### Clade-specific sequence features

We used multiple sequence alignments of the NrnB-like sequences to investigate the uniformity of features within the presumed active site region. Specifically, the DHH domain is known to contain a highly conserved DHH sequence motif and the DHHA1 domain is known to contain a highly conserved GGGH sequence [22]. However, an analysis of residues in these regions revealed differences that are likely to be diagnostic of the different clades (Fig. 1B and C). For example, Clade A exhibits a strong preference for a lysine at position 285 (numbering based on *B. subtilis* NrnB), whereas the corresponding position is strongly conserved as an asparagine in Clade B and is less conserved overall in Clade C. Given the current lack of structural and biochemical data published on NrnB proteins, it is unclear how these sequence preferences might affect enzyme activity, but it is possible that these active site features might influence substrate selectivity.

We then searched for additional features that might differ among the three clades: NrnB-A, NrnB-B, and NrnB-C. While high-resolution structural information is lacking for NrnB proteins, we wanted to investigate whether there might be agreement in the global folds of the different NrnB-containing clades. To address this question, we randomly chose 10 different protein sequences from each clade (Fig. 2A) and then individually subjected the sequences to folding analyses by AlphaFold3 [44]. These data revealed several potentially meaningful trends. For instance, the portions of the proteins that corresponded to the DHH and DHHA1 domains shared a common structural fold. However, the three-dimensional structures predicted by AlphaFold3 exhibited sufficient overall differences that the proteins from all three clades could not be superimposed. Instead, all NrnB-A proteins, including NrnB*_Bs_*, shared a nearly identical structure (Fig. 2A). In contrast, NrnB-B proteins, which are found in Campylobacterota, folded into two separate global conformations (Fig. 2A). Finally, all NrnB-C proteins shared a common overall architecture that was more compact than that of the proteins from the other clades. Therefore, this analysis suggests that the DHH and DHHA1 domains are likely to fold appropriately but that the spacing of these domains relative to one another may differ among the three protein clades. Manual inspection of these proteins revealed that an alpha-helical linker region appears to differ between the three groups of proteins. This linker feature may affect the relative arrangement of the DHH and DHHA1 domains. Prior crystallographic structural analysis of NrnA*_Bs_* showed that there is mobility between the DHHA1 and DHH domains [45]. Specifically, the distance between the DHH and DHHA1 domains varied from 11.9 Å in the ligand-bound state to 19.3 Å in the unbound conformation [45]. These structural dynamics led us to speculate that differences in the linker length could be important for accommodating short versus long RNA substrates. Indeed, AlphaFold-based models suggested the linker length is a key difference between NrnA*_Bs_* and NrnB*_Bs_* [12]. Therefore, we investigated the estimated linker length for all the proteins analyzed in this study (Fig. 2A, inner ring). This structural feature (i.e. linker length) may have a significant impact on substrate recognition, as it changes the relative orientation and spacing of the DHH and DHHA1 domains, which are thought to approach one another in the active site to pinch the nucleotide substrates between the two domains.

A second difference between the three clades corresponded to the length and character of the C-terminus. In our previous analysis of NrnB*_Bs_*, we found that the C-terminal extension present on the DHHA1 domain was needed for processing longer RNA substrates but not for cleavage of short dinucleotide substrates [12]. Specifically, most proteins corresponding to NrnB-A featured a C-terminal extension greater than 100 amino acids in length, whereas most NrnB-B and NrnB-C proteins exhibited shorter C-terminal regions (<50 amino acids). The portion of the C-terminal region that was contained in all three clades demonstrated strong sequence preferences for certain amino acids in NrnB-A (Fig. 2B and C); however, the extended region that is unique to NrnB-A also appeared to be highly conserved. An electrostatic surface potential map of this region of NrnB*_Bs_* shows that these residues create a positively charged patch on the surface of NrnB-A proteins (Fig. 2B). Therefore, NrnB-A proteins appear to feature a C-terminal-extension sequence common among that clade. In contrast, the NrnB-B and NrnB-C proteins lacked this extension sequence and showed only moderate conservation in the part of the C-terminal region shared in all three clades (Fig. 2C). From these aggregate data, we conclude that the three clades show global structural features that distinguish them from one another.

### NrnB protein candidates degrade RNAs of varying lengths *in vitro*

It is difficult to predict the functions and substrate preferences of standalone DHH–DHHA1 homologs. Therefore, in this study, we investigated the substrate preferences of purified proteins representing each of the three clades of NrnB homologs. Specifically, we purified *B. licheniformis* NrnB (NrnB*_Bl_*) to represent clade NrnB-A, *B. thuringiensis* NrnB (NrnB*_Bt_*) to represent clade NrnB-C, and *H. pylori* NrnB (NrnB*_Hp_*) to represent clade NrnB-B.

**Figure 3.**
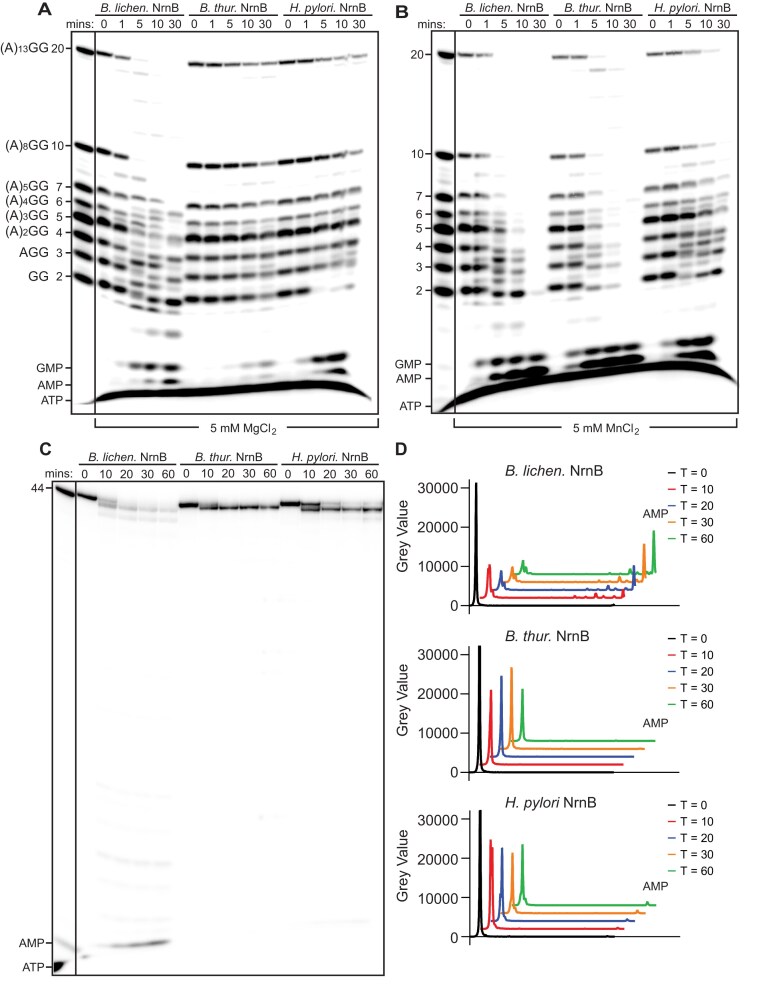
NrnB proteins degrade RNAs of varying lengths *in vitro*. (**A**) Trace amounts of 5′ ^32^P-radiolabeled RNA molecules that were 2–7, 10, and 20 nucleotides in length were mixed and simultaneously incubated with 50 nM of purified NrnB*_Bl_*, NrnB*_Bt_*, or NrnB*_Hp_* in the presence of 5 mM MgCl_2_, or (**B**) 5 mM MnCl_2_. (**C**) One micromolar of a monophosphorylated, unstructured 44-mer RNA molecule containing a trace amount of 5′-^32^P-radiolabeled 44-mer RNA were incubated with 100 nM of NrnB*_Bl_*, NrnB*_Bt_*, or NrnB*_Hp_*. Samples were removed at time intervals and analyzed by urea-denaturing 20% PAGE. (**D**) Line trace quantification of the indicated time points in panel (C). At least three experimental replicates were used for all experiments in this figure.

We conducted time-course RNase cleavage assays by simultaneously incubating trace amounts of 5′-^32^P-radiolabeled RNAs (2–7, 10, and 20 nucleotides in length) with 50 nM purified proteins and analyzed cleavage patterns after incubating in either magnesium or manganese as a catalytic metal ion (Fig. 3A and B). In the presence of manganese, all proteins could fully process RNA substrates to monoribonucleotides; therefore, all NrnB homologs possessed both exoribonucleolytic and nanoRNase activities. However, the proteins were less active overall and showed differences in their substrate preferences when incubated with magnesium. In the presence of magnesium, NrnB*_Bl_* was active against substrates of all lengths, whereas NrnB*_Bt_* and NrnB*_Hp_* exhibited a clear preference for short RNAs. It is noteworthy that NrnB*_Hp_* processed the diribonucleotide pGpG to near completion in 5 min in the presence of either manganese of magnesium, whereas it showed only minimal activity against any substrate longer than the dinucleotides in the presence of magnesium (Fig. 3A and B). Together, these data suggest that manganese either alters the substrate length accommodation or increases the overall turnover rate, such that processing of longer RNAs becomes observable.

We also found that NrnB*_Bt_* and NrnB*_Hp_* proteins processed dinucleotides even at low concentrations (10 nM; Supplementary Fig. S2), while processing of longer substrates required at least five-fold more protein (Fig. 3, Supplementary Fig. S2). Indeed, at 100 nM NrnB*_Bl_* was active against an unstructured 44-mer RNA, whereas NrnB*_Bt_* and NrnB*_Hp_* showed only a small amount of cleavage from the 3′ terminus of the 44-mer substrate (Fig. 3C). Taken altogether, these data suggest that NrnB*_Bl_* can act as a general exoribonuclease as well as a nanoRNase, while NrnB*_Hp_* and NrnB*_Bt_* are likely to exhibit a preference for diribonucleotides under physiological conditions.

### NrnB proteins generally initiate RNA degradation from the 3′ terminus

**Figure 4.**
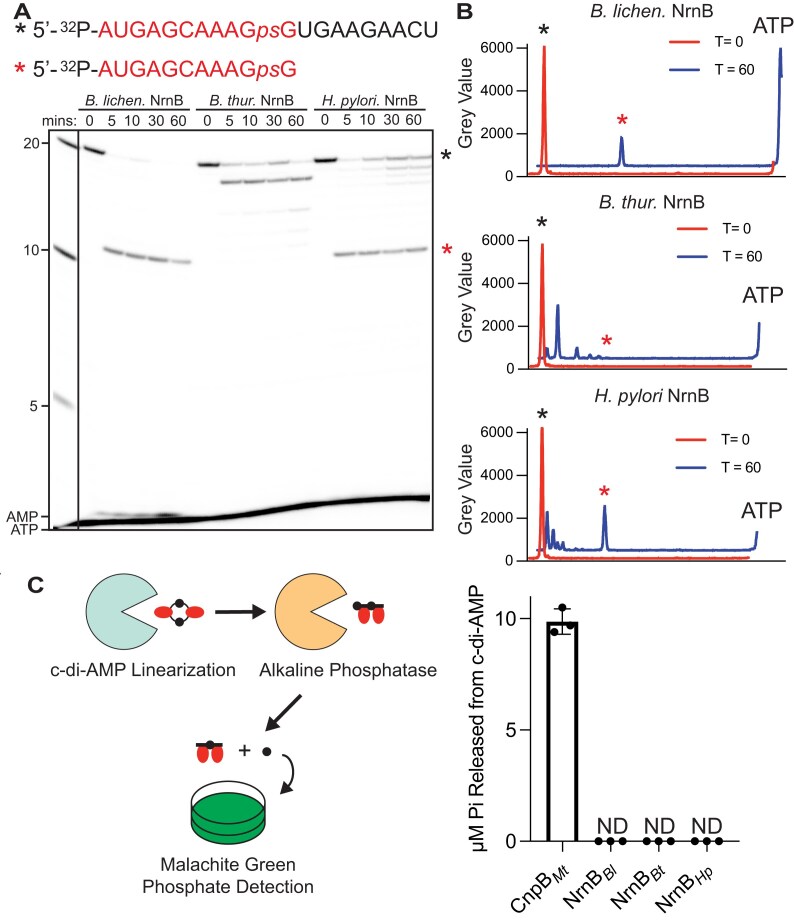
NrnB proteins act on RNAs from the 3′ terminus but do not process cyclic di-AMP substrates. (**A**) Trace amounts of a 5′ ^32^P-radiolabeled 20-mer RNA containing an internal phosphorothioate linkage at the 10th residue was subjected to cleavage by 400 nM of NrnB*_Bl_*, NrnB*_Bt_*, or NrnB*_Hp_*. Aliquots were removed from reactions and quenched in 150 mM EDTA and 4 M urea. Degradation products were resolved by 20% denaturing PAGE. (**B**) A representative line trace quantification of the *T* = 0- and *T* = 60-min point degradation products in panel (A). (**C**) NrnB-like proteins were analyzed indirectly for c-di-AMP processing based on a coupled enzyme reaction where c-di-AMP linearization results in exposed 5′ phosphate, which is readily liberated by alkaline phosphatase. The release of inorganic phosphate was quantified using the Sigma–Aldrich^®^ Malachite Green Phosphate Assay Kit (MAK307). Five hundred nanomolar of the respective protein was incubated with 20 µM of c-di-AMP in the presence of 10 U of alkaline phosphatase in a 40 µl reaction volume. At least three experimental replicates were used for all experiments in this figure. “N.D.” indicates that no data were detected.

Previous studies have shown that NrnA*_Bs_* and NrnB*_Bs_* degrade RNA in different directions. Where NrnA*_Bs_* utilizes a 5′-3′ exoribonucleolytic mechanism, NrnB*_Bs_* instead degrades RNAs using a 3′-5′ polarity [12, 13]. To conclusively determine the polarity of NrnB*_Bl_*, NrnB*_Bt_*, and NrnB*_Hp_*, we assessed the cleavage of trace amounts (~40 nM) of 5′-^32^P-20-mer RNA containing an internal non-cleavable phosphorothioate linkage at the 10th position of the RNA. We reasoned that if the NrnB proteins employed a 5′-3′ mechanism, we would only observe the removal of the 5′-radiolabeled mononucleotide ^32^P-AMP. In contrast, if NrnB homologs degrade the 5′-^32^P-20-mer RNA from the 3′ terminus, cleavage should be halted as the enzymes reach the internal phosphorothioate linkage (5′-^32^P-AUGAGCAAAG*ps*GUGAAGAACU), resulting in a degradation intermediate corresponding to an 11-mer RNA (5′-^32^P-AUGAGCAAAG*ps*G). In our initial experiments for assessing the polarity of the NrnB proteins, we observed that NrnB*_Hp_* and NrnB*_Bt_* did not exhibit robust activity against the phosphorothioate containing substrate, so we increased the concentration of protein relative to other assays in this analysis. When we incubated 400 nM NrnB*_Bl_* with 5′-^32^P-20-mer RNA containing the internal phosphorothioate linkage, we observed a rapid degradation product that corresponded to 5′-^32^P-AUGAGCAAAG*ps*G. Interestingly, we also observed a moderate accumulation of ^32^P-AMP monoribonucleotides at later time points (Fig. 4A). From this, we speculate that NrnB*_Bl_* might slowly process through the phosphorothioate linkage at this protein concentration. Alternatively, accumulation of ^32^P-AMP could also potentially result from a minor amount of cleavage activity from the 5′ terminus. However, the cleavage intermediates that generally result from NrnB*_Bl_* reactions (*e.g*. Fig. 3) are consistent overall with processing from the 3′ terminus; therefore, we speculate that 3′-5′ exonucleolytic activity is significantly preferred by NrnB*_Bl_*. Incubation of 400 nM NrnB*_Bt_* with 5′-^32^P-20-mer RNA containing the internal phosphorothioate linkage primarily revealed degradation intermediates that corresponded to the removal of two nucleotides from the 3′ end, thereby generating 5′-^32^P-AUGAGCAAAG*ps*GUGAAGAA (Fig. 4A). We do not observe any ^32^P-AMP monoribonucleotides accumulate in reactions with NrnB*_Bt._* Together, these data provide additional support that this protein strongly disfavors longer RNA substrates. Incubation of 400 nM NrnB*_Hp_* with the 5′-^32^P-AUGAGCAAAG*ps*GUGAAGAA resulted in the 11-mer degradation product resulting from the phosphorothioate site. These aggregate data strongly suggest that the NrnB-like protein homologs from *B. licheniformis, B. thuringiensis*, and *H. pylori* all process RNAs using a 3′-5′ exonucleolytic mechanism. Therefore, we conclude that polarity is a diagnostic biochemical criterion for distinguishing between NrnA and NrnB proteins contained within the DHH–DHHA1 protein superfamily.

### NrnB protein candidates do not act on c-di-AMP

Multiple studies have demonstrated that certain DHH–DHHA1 protein family members process c-di-AMP into monoribonucleotides [2, 3, 24, 25]. Since c-di-AMP is a possible substrate for this protein family, we tested whether NrnB*_Bl_*, NrnB*_Bt_*, or NrnB*_Hp_* exhibited activity against the c-di-AMP signaling molecule. To rapidly assess c-di-AMP phosphodiesterase cleavage activity, we devised a quick nonradioactive method where we included alkaline phosphatase in the reaction mixtures. In this reaction scheme, we observed that c-di-AMP does not contain phosphates recognizable by alkaline phosphatase. However, when c-di-AMP is linearized to di- or monoribonucleotides, alkaline phosphatase can liberate the 5′ phosphate, which can be detected by the Malachite-Green-based absorbance assay. In these assays, we incubated 500 nM of purified NrnB*_Bl_*, NrnB*_Hp_*, NrnB*_Bt_*, or CnpB*_Mt_* with 20 µM c-di-AMP in the presence of excess alkaline phosphatase for 30 min prior to measuring inorganic phosphate release. We observed that ~10 µM of free phosphate was released in reactions containing the bona fide c-di-AMP phosphodiesterase CnpB*_Mt_*. However, our analysis did not reveal any processing of c-di-AMP by NrnB*_Bl_*, NrnB*_Hp_*, or NrnB*_Bt_* after a 30-min incubation (Fig. 4C).

### Processing of dinucleotides *in vivo* by NrnB protein candidates

**Figure 5.**
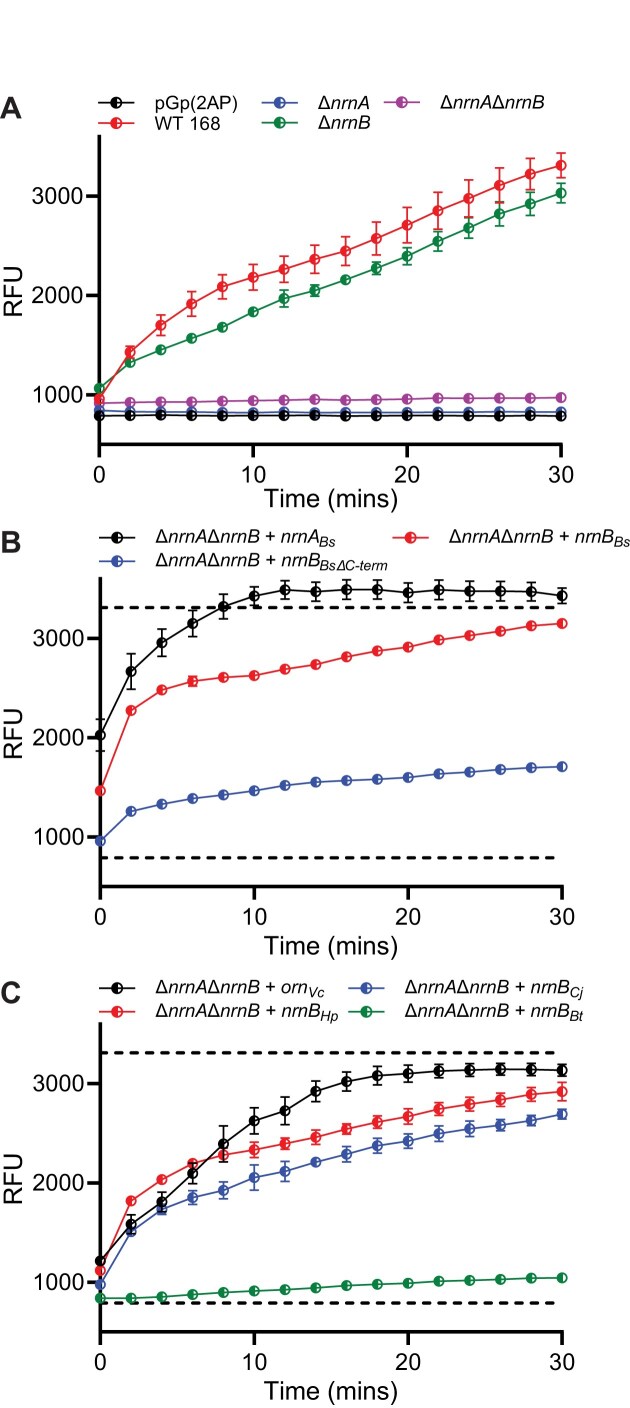
NrnB proteins from Clade B display *ex vivo* dinuclease activity against pGp(2AP). (**A**) *Bacillus subtilis* cellular extracts for WT 168, ∆*nrnA*, ∆*nrnB*, ∆*nrnA*∆*nrnB*, (**B**) IPTG inducible complementation strains of ∆*nrnA*∆*nrnB* with *nrnA_Bs_, nrnB_Bs_, nrnB_Bs∆C-term._*, or (**C**) *orn_Vc_, nrnB_Hp_, nrnB_Cj._*, and *nrnB_Bt._* were incubated with 5 µM pGp(2AP) in a plate reader. (2AP) release was monitored every 2 min using the excitation wavelength of 315 and emission wavelength of 375. Dashed lines in panels (B) and (C) indicate the maximum fluorescence signal observed for the positive control WT 168 and the minimum fluorescence signal for the negative control pGp(2AP). At least three experimental replicates were used for all experiments in this figure.

While purified NrnB proteins exhibited activity against short RNAs, we sought to determine whether expression of the corresponding *nrnB* genes could complement a dinuclease-deficient phenotype in *ex vivo* experiments. Specifically, lysates were prepared from mutant *B. subtilis* strains (e.g. Δ*nrnA*, Δ*nrnB*, and Δ*nrnA/*Δ*nrnB*). Lysates were also prepared from complementation strains wherein inducible copies of different *nrnA, nrnB*, or *orn* genes were integrated into the genome of the *B. subtilis* Δ*nrnA*Δ*nrnB* strain. The dinucleotide substrate, which was comprised of a guanosine monophosphate followed by a 2-aminopurine (2AP) monophosphate, was then added to the lysates and dinucleotide cleavage was measured via fluorescence spectrophotometry as release of 2AP. Lysates from wild-type cells showed activity against pGp(2AP); however, deletion of *nrnA* led to complete loss of dinucleotide cleavage (Fig. 5A). This phenotype could be complemented by expression of *B. subtilis nrnA* or, to a lesser extent, *B. subtilis nrnB* (Fig. 5B). Cleavage of dinucleotide was also restored by expression of a known dinuclease gene, *V. cholera orn* (Fig. 5C). Expression of either *nrnB_Hp_* or *nrnB_Cj_* restored processing of dinucleotides, although expression of *nrnB_Bt_* only led to a minimal increase in cleavage (Fig. 5C). From these data, we conclude that the NrnB*_Hp_* and NrnB*_Cj_* homologs are active against dinucleotides *in vivo*.

### *Helicobacter pylori* and *B. thuringiensis* NrnB show general exoribonuclease activity but preferentially degrade short RNAs

For a closer inspection of the substrate preferences exhibited by *H. pylori* NrnB, we individually incubated 1 µM RNAs of varying lengths (2–7, 10, or 15 nucleotides) with 50 nM purified NrnB*_Hp_*. The products of those reactions were then resolved by urea-denaturing PAGE. This analysis revealed that dinucleotide and trinucleotide substrates were rapidly and fully processed to mononucleotides (Supplementary Fig. S3). In contrast, NrnB*_Hp_* showed less activity against longer RNA substrates under these reaction conditions (Supplementary Fig. S3). The degradation intermediates that are readily observable in the processing of RNAs 4–10 nucleotides in length are consistent with a 3′-5′ exonucleolytic mechanism. Together, these data suggest that while NrnB*_Hp_* has the theoretical capacity to act as a general exoribonuclease, akin to NrnB*_Bs_*, it exhibits an NrnA-like preference for short RNAs 2–4 nucleotides in length. Therefore, we speculate that NrnB*_Hp_* likely functions as a true nanoRNase enzyme.

We also individually incubated 1 µM RNAs of varying lengths (2–7, 10, or 15 nucleotides) with 50 nM purified NrnB*_Bt_*. NrnB*_Bt_* rapidly processed 2-mer RNAs. While it also processed some of the other RNAs to mononucleotides, it showed declining activity against the longer substrates (Supplementary Fig. S4). Therefore, NrnB*_Bt_* exhibits an NrnA-like preference for short RNAs 2–4 nucleotides in length, while also displaying the potential to act on longer RNA substrates at higher concentrations.

### Cellular lysates containing NrnB protein homologs can enhance the rate of 20-mer processing

Our prior investigation of *B. subtilis* NrnB showed a substrate profile that differed modestly from that displayed herein by NrnB*_Hp_* or NrnB*_Bt_* [12]. Instead, the NrnB*_Bs_* substrate profile more closely resembles that of NrnB*_Bl_* in the current study. This is notable, as they are both members of the NrnB-A clade, suggesting that the substrate preferences shown by NrnB*_Bs_* and NrnB*_Bl_* are broadly representative of this overall group of proteins. Specifically, we previously showed that NrnB*_Bs_* can exonucleolytically degrade longer RNAs (6–20 nucleotides in length) to near completion by the 30-min time point using the same assay conditions as described herein. One notable feature of the NrnB-A clade is the presence of a longer C-terminal extension that follows the DHHA1 domain (Fig. 2A and B). While the remaining two clades showed a C-terminal sequence of <50 amino acids in length, proteins in the NrnB-A clade featured a positively charged C-terminal sequence that was greater than 110 amino acids long. Since the NrnB-A clade proteins (NrnB*_Bs_* and NrnB*_Bl_*) perform best against longer substrates, we hypothesized that the C-terminal extension is a necessary accessory for degradation of long RNAs. To further test this hypothesis, we investigated the role of the C-terminus for an additional representative of the NrnB-A clade, *B. megaterium* NrnB, alongside an additional representative of the NrnB-B clade, *C. jejuni* NrnB. We reasoned that these proteins should behave similarly to the proteins in their respective clades.

**Figure 6.**
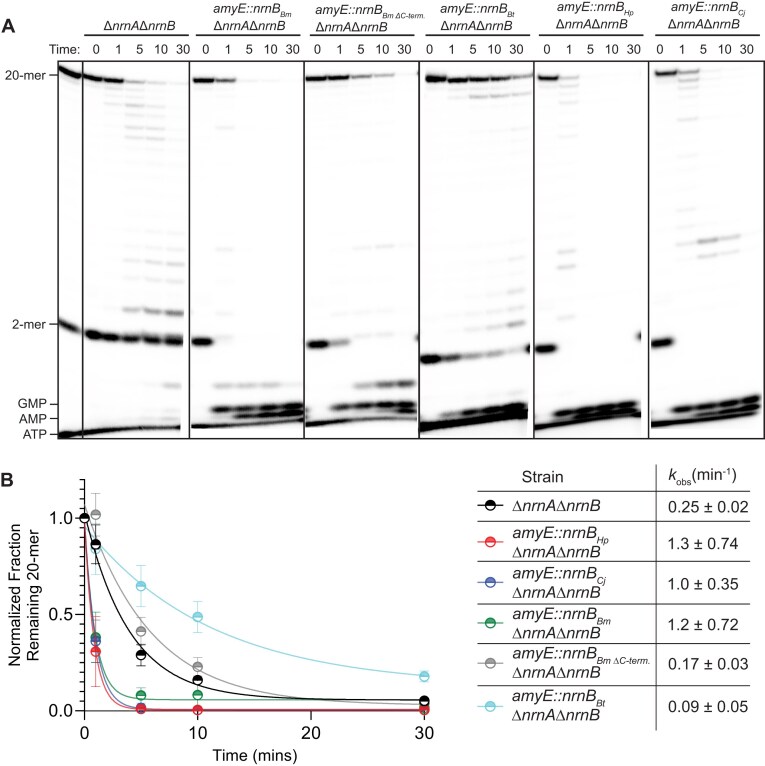
NrnB proteins degrade diribonucleotides in ∆*nrnA*∆*nrnB B. subtilis* cellular lysates. (**A**) Cellular lysates for ∆*nrnA*∆*nrnB* or ∆*nrnA*∆*nrnB* that had been complemented with an IPTG inducible copy of *nrnB_Bm_, nrnB_Bm∆C-term_, nrnB_Bt_, nrnB_Hp_*, or *nrnB_Cj_*, which were induced with 250 µM IPTG at an OD_600_ = 1 and grown for a further 40 min at 37°C prior to harvesting the cells. To the cellular lysates, trace amounts of both a 5′-^32^P-radiolabeled RNA diribonucleotide (pGpG) and a 20-mer RNA were simultaneously added to the reactions in a buffer containing 10 mM MgCl_2_ and 50 µM MnCl_2_. (**B**) The normalized radioactive intensity of the initial substrate depletion normalized to the first time point plotted as an average and SD of three independent experiments. At least three experimental replicates were used for all experiments in this figure.

For *ex vivo* experiments, we generated whole cell lysates of *B. subtilis* ∆*nrnA*∆*nrnB* or strains of *B. subtilis* ∆*nrnA*∆*nrnB* that also contained single-gene integration of an ectopic IPTG-inducible copy of *nrnB_Bt_*, *nrnB_Bm_, nrnB_Pm_*_∆C-term._, *nrnB_Hp_*, or *nrnB_Cj_*. The *nrnB_Bm_*_∆C-term._ mutant construct was created by deleting the residues (306–395) corresponding to the C-terminal extension subdomain that follows the DHHA1 domain. The cellular lysates were simultaneously incubated with trace amounts of 5′-radiolabeled diribonucleotide pGpG and a 5′-radiolabeled 20-mer. The degradation products from these lysate experiments were resolved by 20% denaturing PAGE. In agreement with our previous analysis, we found that the diribonucleotide pGpG remained unprocessed when incubated with ∆*nrnA*∆*nrnB B. subtilis* lysate (Fig. 6A), highlighting a need for NrnA*_Bs_* for processing dinucleotides. However, the 20-mer RNA was processed over time by the ∆*nrnA*∆*nrnB B. subtilis* lysate to near completion. Next, we added IPTG to forcibly express *nrnB_Bt_*, *nrnB_Bm_, nrnB_Bm_*_∆C-term._, *nrnB_Hp_*, or *nrnB_Cj_* and prepared cell lysates to repeat the *ex vivo* RNA cleavage assays. Induction of any of the five *nrnB* genes resulted in the rapid and complete processing of diribonucleotides, supporting their role as nanoRNases (Fig. 6A). However, degradation of the 20-mer was not enhanced equally by all these genes. Expression of *nrnB_Bt_* did not enhance the rate of 20-mer degradation, while expression of either *nrnB_Hp_* or *nrnB_Cj_* resulted in enhanced processing of the 20-mer. Degradation of the 20-mer RNA was also enhanced by expression of *nrnB_Bm_*(Fig. 6A and B). Yet, this enhancement effect was lost when the C-terminal extension was deleted from *nrnB_Bm_*. Notably, the extract containing *nrnB_Bm_*_∆C-term_ still resulted in rapid and complete processing of the dinucleotide substrate.

### NrnB shows a preference for diribonucleotide substrates

**Figure 7.**
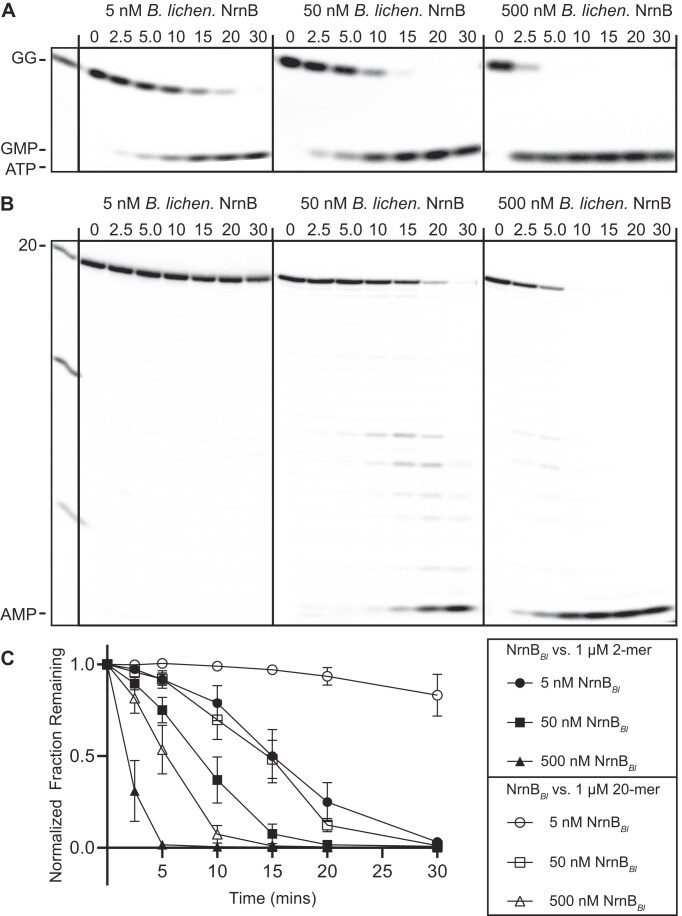
*Bacillus licheniformis* NrnB degrades dinucleotides at low protein concentrations (**A**–**C**). One micromolar of RNA molecules 2 or 20 nucleotides in length containing a trace amount of 5′-^32^P-radiolabeled RNA were incubated with purified NrnB*_Bl_* at enzyme concentrations ranging from 5 to 500 nM in a reaction containing 25 mM Tris–HCl (pH 8.0), 100 mM NaCl, 5 mM MnCl_2_. Samples were removed at time intervals and analyzed by urea-denaturing 20% PAGE. (**C**) Quantification of the normalized radioactive intensity of the initial substrate depletion over time plotted as the average and SD of three independent experiments in panels (A) and (B). Aliquots were removed from reactions and quenched in 150 mM EDTA and 4 M urea. Degradation products were resolved by 20% denaturing PAGE. At least three experimental replicates were used for all experiments in this figure.

**Figure 8.**
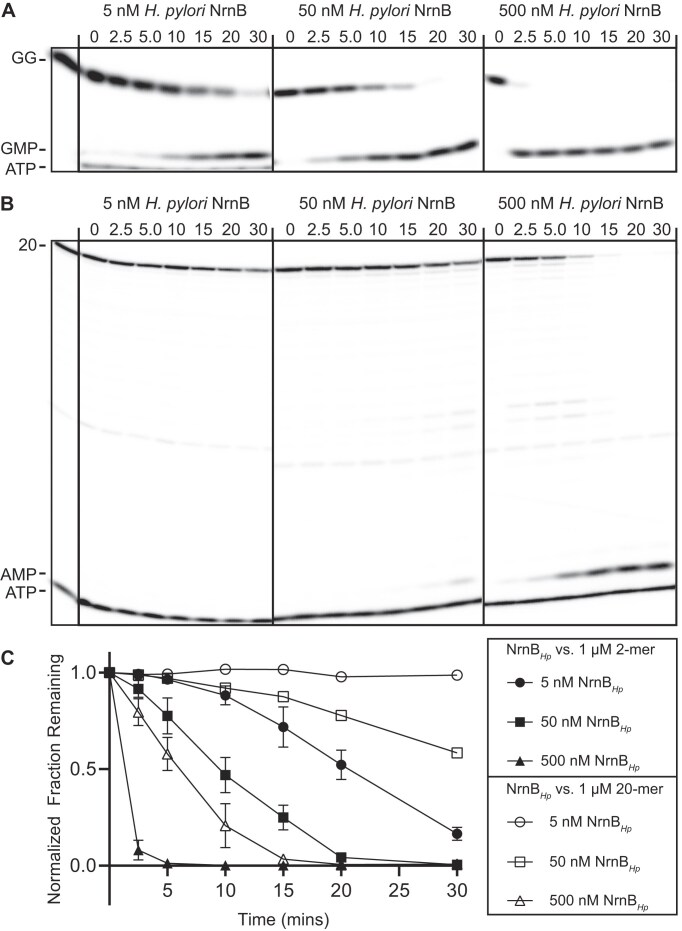
*Helicobacter pylori* NrnB degrades dinucleotides at low protein concentrations (**A**–**C**). One micromolar of RNA molecules 2 or 20 nucleotides in length containing a trace amount of 5′-^32^P-radiolabeled RNA were incubated with purified NrnB*_Hp_* at enzyme concentrations ranging from 5 to 500 nM. Samples were removed at time intervals and analyzed by urea-denaturing 20% PAGE. (**C**) Quantification of the normalized radioactive intensity of the initial substrate depletion over time plotted as the average and SD of three independent experiments in panels (A) and (B). Aliquots were removed from reactions and quenched in 150 mM EDTA and 4 M urea. Degradation products were resolved by 20% denaturing PAGE. At least three experimental replicates were used for all experiments in this figure.

There are at least two possibilities for NrnB proteins: (i) they show equal preference between short and long RNA substrates or (ii) they demonstrate activity against long RNA substrates but show a strong preference for short RNA substrates. The latter model would suggest that these proteins have evolved to function as nanoRNases, while the former model would suggest that they act as general exoribonucleases. Our initial assessment (Fig. 3) of NrnB*_Hp_* used ~40 nM of radiolabeled RNA substrates with a protein concentration of 50 nM. To further expand this protein substrate ratio, we incubated 5, 50, or 500 nM NrnB with 1 µM of either the diribonucleotide pGpG or 20-mer RNA, each containing trace amounts of 5′-^32^P-radiolabeled RNA. We conducted these assays using either purified NrnB*_Bl_* (Fig. 7), to represent clade NrnB-A, or purified NrnB*_Hp_* (Fig. 8), to represent clade NrnB-B. In both instances, even lower amounts of protein resulted in detectable cleavage of diribonucleotide, whereas processing of the 20-mer RNA was only observed at higher protein concentrations. Therefore, we conclude that NrnB*_Bl_* and NrnB*_Hp_*, and by extension the other proteins contained within their clades, respectively, act primarily as nanoRNases, despite having the potential for processing longer RNAs.

## Discussion

Of the four major classes of bacterial nanoRNases, the NrnB proteins have been the least studied. Although structural data have been acquired for representatives of the Orn, NrnC, and NrnA classes of proteins, no high-resolution three-dimensional structures have been published for an NrnB protein. Furthermore, only the *B. subtilis* NrnB homolog has been biochemically examined for its activity. Therefore, in this study, we searched broadly for bacterial NrnB homologs and investigated their phylogenetic distributions. While our analysis identified NrnB homologs from all three domains of life (Supplementary Fig. S1), with a particular representation among Bacillota and Campylobacterota, we focused our bioinformatic analysis on the phylogenetic branch that contained the *B. subtilis* NrnB sequence. This branch includes three subclades of NrnB homologs, which we refer to as NrnB-A, NrnB-B, and NrnB-C, which could be further identified based on key signature sequence features (Fig. 1). Representative proteins from each NrnB cluster were purified and examined for RNase activity using biochemical assays. Biochemical analyses revealed two key observations: (i) processing of RNA substrates in the 3′-5′ direction is a universal feature of bacterial NrnB proteins and (ii) all bacterial NrnB proteins show an ability to process nanoRNAs (between 2 and 4 nucleotides in length).

The NrnB-A clade proteins, which includes *B. subtilis* NrnB, were primarily found in Bacillota. We previously showed that NrnB*_Bs_* acts as a unique 3′-5′ exonuclease that acts against short and long RNA substrates [12]. However, while we have elucidated some of the biochemical properties of NrnB*_Bs_*, their functional role(s) in cells are less clear. The ∆*nrnA*∆*nrnB* double mutant exhibits a dramatic 99% reduction in sporulation efficiency, as compared to wild-type cells [12]. Moreover, NrnB*_Bs_* is specifically expressed within the forespore during late stages of sporulation. Therefore, it is possible that a general exonuclease that can function against small and large RNA substrates is required during bacterial sporulation and/or germination. If so, we would predict that other sporulating bacteria either encode an NrnB homolog or express an alternative, and perhaps unknown, exonuclease. Indeed, manual inspection of the DNA region upstream of the gene encoding NrnB*_Bl_* also appeared to contain a promoter sequence that resembles the Sigma G-activated promoter found upstream of *B. subtilis nrnB*. While it remains to be determined whether sporulation promoters can be identified upstream of the other genes encoding NrnB-A clade proteins, we tentatively speculate that Clade A proteins are likely to be utilized for an unknown function during sporulation. However, a corollary of that statement is that these sporulating bacteria would also require a separate housekeeping nanoRNase gene, such as NrnA, for their global processing of short RNAs. Inspection of *B. licheniformis* and *B. megaterium* genomes revealed that both appear to encode for a separate *nrnA* gene. Therefore, it is possible that the bacteria represented in Clade A use NrnA for housekeeping nanoRNase activity while using NrnB for an unknown specialized purpose during sporulation, although further bioinformatics analyses would be required to support that hypothesis.

Our data also revealed a relationship between the C-terminal extension found in the NrnB-A clade proteins and their substrate selection. The NrnB-A proteins all featured a long, conserved C-terminal extension (Fig. 2) and the representative proteins that we characterized herein (NrnB*_Bs_*, NrnB*_Bl_*, and NrnB*_Pm_*) proved to be highly active against long RNA substrates (Fig. 6). In contrast, the NrnB-B and NrnB-C clade proteins lacked this C-terminal extension and exhibited a preference for short RNA substrates (Fig. 3). Therefore, we conclude that the long C-terminal extension is a diagnostic criterion among bacterial NrnB proteins for general 3′-5′ exoribonuclease activity.

Our bioinformatics analysis indicated that the NrnB-B clade was restricted to Campylobacterota (Fig. 1). An informal survey of a few representative members of the NrnB-B clade suggested that their genomes are not likely to encode for any of the other nanoRNase classes, Orn, NrnA, or NrnC. We speculate that the NrnB homologs found in the genomes of Campylobacterota, such as those encoded by *H. pylori* and *C. jejuni*, act as true nanoRNases, the only phosphodiesterases in these bacteria that can process very short RNAs. However, the suite of enzymes involved in global RNA degradation are still generally understudied in *H. pylori* and *C. jejuni*; therefore, it is still possible that the NrnB-like protein that they encode acts as a true nanoRNase, a global exonuclease, or both. Currently, none of the known global exoribonucleases (e.g. RNase J, PNPase, RNase R) have been shown to participate in short RNA processing in *H. pylori* or *C. jejuni*, or in *in vitro* assays conducted with protein homologs from other organisms [46–48]. Resolving the biochemical activities of NrnB proteins is important as it may have biomedical implications. The Orn nanoRNase is essential in Gammaproteobacteria that have been investigated, due to a toxic accumulation of dinucleotides that is triggered by depletion of Orn [10]. Similarly, a recent transposon sequencing analysis of *C. jejuni* NCTC 11168 provided evidence that the gene encoding NrnB (locus tag: Cj0881c) is also likely to be essential for the growth of this organism [7]. Therefore, NrnB might represent a druggable target, if essentiality indeed proves to be a general feature of the Clade B NrnB proteins.

The NrnB-C clade proteins were also specifically found in genomes of Bacillota (Fig. 1). Similar to the NrnB-B clade proteins, the NrnB-C proteins exhibited a preference for shorter nucleic acid substrates (Supplementary Figs S3 and S4). These proteins also lacked the C-terminal extension found in the NrnB-A clade. From these aggregate data, we hypothesize that the NrnB-C proteins act as a true global nanoRNase for the bacteria that produce them. We speculate that the bacterial genomes encoding for NrnB-C proteins are unlikely to encode for any of the remaining classes of nanoRNases. However, further bioinformatics analyses are needed to test that hypothesis.

Recent publications have given key insights into the general properties of Orn, NrnA, and NrnC nanoRNases [1]. These data showed that Orn and NrnC are 3′-5′ exonucleases that exhibit a preference for dinucleotide substrates. In contrast, the NrnA proteins that have been characterized have been found to be 5′-3′ exonucleases that act on oligonucleotides from 2–4 nucleotides in length. However, our data herein argue that NrnB proteins exhibit striking differences from NrnA, despite their shared evolutionary history and similar global protein folds. Our data demonstrate that a defining feature of NrnB proteins is that they act as general 3′-5′ exonucleases that exhibit a substrate range that is broader than NrnA proteins. All NrnB proteins are likely to act as nanoRNases, capable of substituting for the loss of NrnA, and capable of processing short RNAs under physiological conditions. However, we also speculate that subsets of NrnB homologs are likely to act as general exoribonucleases, capable of cleaving long RNAs as well as short RNAs. Overall, these data significantly improve the functional annotation of putative nanoRNase genes.

## Supplementary Material

gkaf1384_Supplemental_File

## Data Availability

Mendeley Data is an open-source depository of primary data (https://data.mendeley.com/my-data/). Upon publication, all data will be accessible with a public accession number doi: 10.17632/xmk4tx8bjp.1.
