# Supplementary material for: Functional analyses of bacterial NanoRNase B proteins reveals defining features of this enzyme family
Source: Nucleic Acids Res. 2025 Dec 17;53(22):gkaf1384. doi: 10.1093/nar/gkaf1384 (PMC12709177; doi:10.1093/nar/gkaf1384)
Supplement: gkaf1384_Supplemental_File [file gkaf1384_supplemental_file.pdf]

SUPPLEMENTARY FIGURES

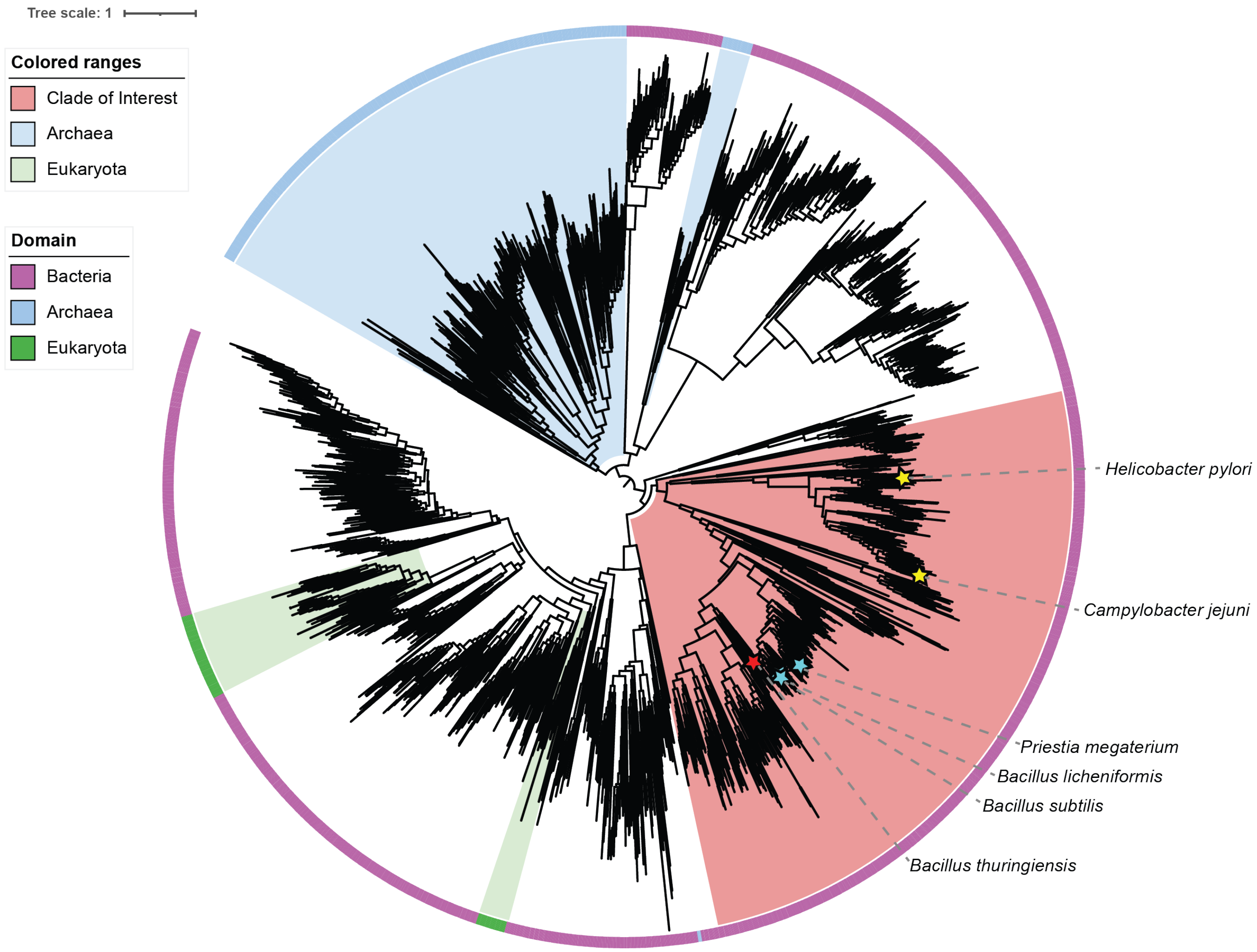

Figure S1. Phylogenetic analysis of *nrnB* across prokaryotic and eukaryotic domains. A circular display of a phylogenetic tree of NrnB sequences. Notable species including *Helicobacter pylori*, *Campylobacter jejuni*, *Priestia megaterium*, *Bacillus licheniformis*, *Bacillus subtilis*, and *Bacillus thuringiensis* are marked with star symbols to indicate their positions within the tree. Branches are color-coded by domain: Archaea (blue), Bacteria (purple), and Eukaryota (green). The bacterial clade of interest is highlighted in red.

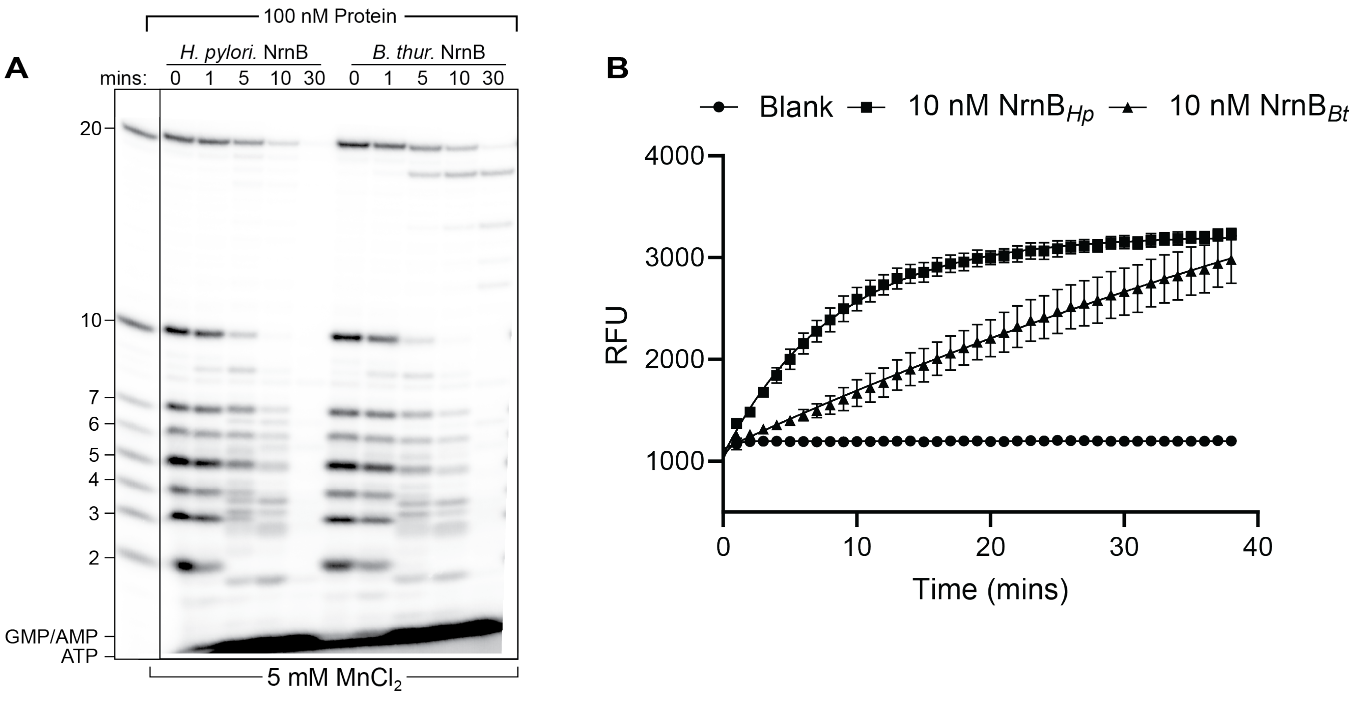

Figure S2. NrnB$_{Hp}$ and NrnB$_{Bt}$ processes RNAs of varying lengths and show robust processing of pGp(2AP). (A) Trace amounts of 5' ^{32}P-radiolabeled RNA molecules that were 2-7, 10, and 20 nucleotides in length were mixed and simultaneously incubated with 100 nM of purified NrnB$_{Hp}$ or NrnB$_{Bt}$ in the presence of 5 mM $MnCl_2$. Aliquots were removed from reactions and quenched in 150 mM EDTA and 4 M urea. Degradation products were resolved by 20% denaturing PAGE. (B) 5 µM of pAp (2AP) was subjected to cleavage by 10 nM of NrnB$_{Hp}$ or NrnB$_{Bt}$ in the presence of 5 mM $MgCl_2$ and 500 µM $MnCl_2$ where the fluorescence corresponding to the release of free (2AP) was measured continuously every two minutes using ex. 315 and em. 375 on a Spectramax M5 plate reader.

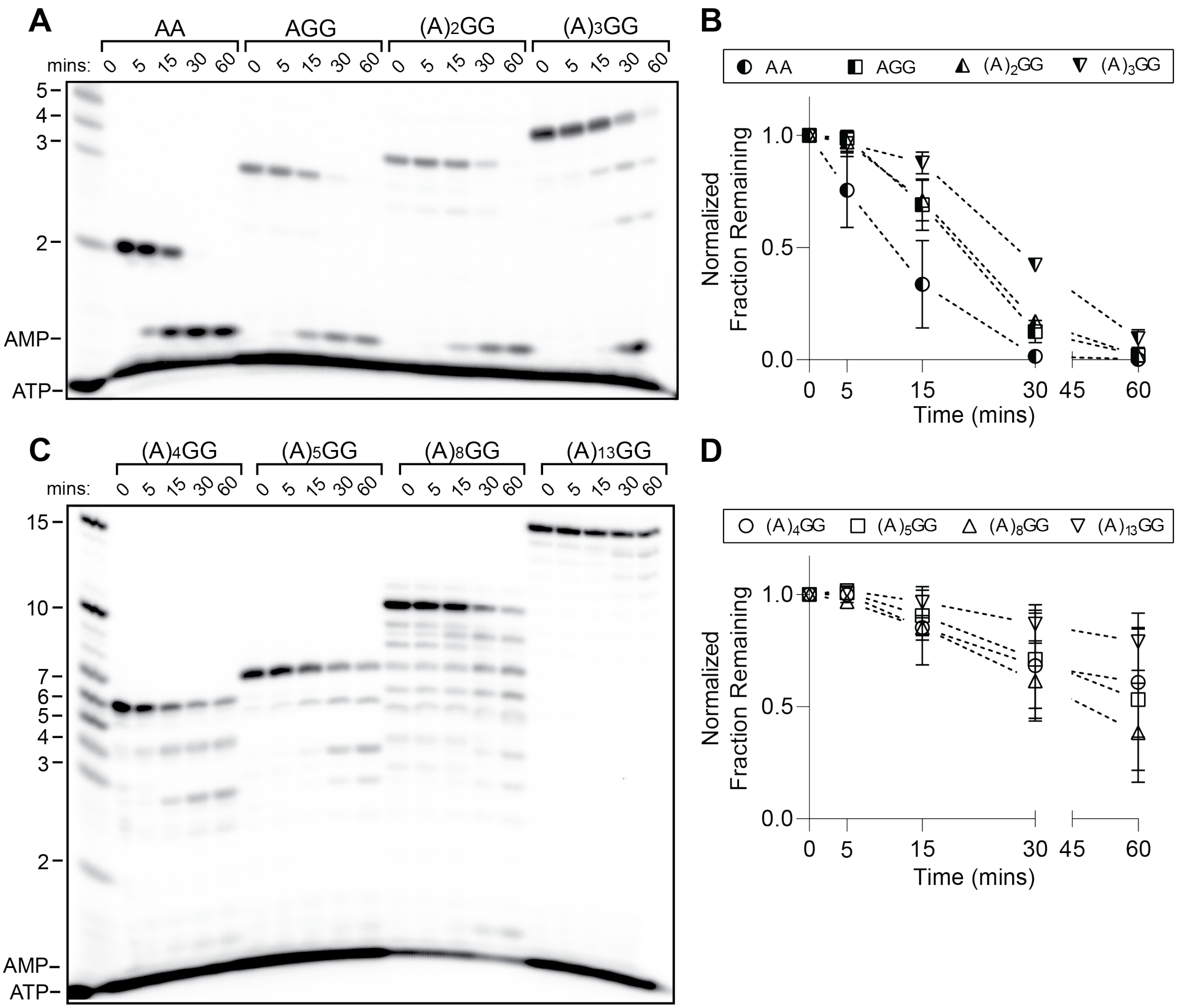

Figure S3. *H. pylori* NrnB preferentially hydrolyzes short RNAs 2-4 nucleotides in length. (A, C) Native RNA molecules 2-7, 10, and 15 nucleotides in length were incubated at a final concentration of 1 μM with 50 nM of purified NrnB$_{Hp}$ and 5 mM manganese. These reactions also contained a trace amount of 5'-^{32}P-radiolabeled RNA. Samples were removed at time intervals and analyzed by urea-denaturing 20% PAGE. (B, D) Quantification of the normalized radioactive intensity of the initial substrate depletion over time plotted as the average and SD of 3 independent experiments in (A, C). Aliquots were removed from reactions and quenched in 150 mM EDTA and 4 M urea. Degradation products were resolved by 20% denaturing PAGE.

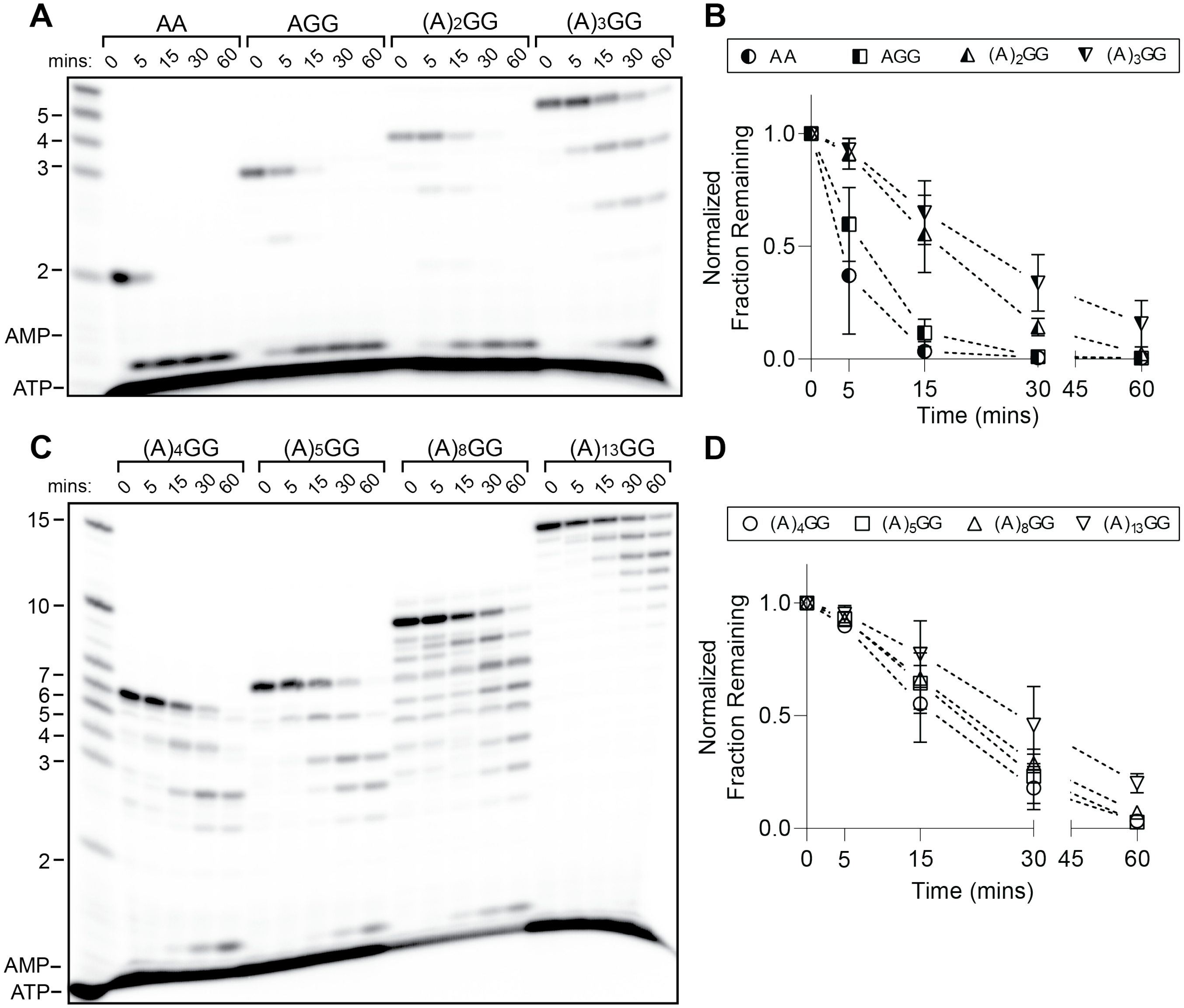

Figure S4. *B. thuringiensis* NrnB preferentially hydrolyzes short RNAs 2-4 nucleotides in length. (A, C) RNA molecules 2-7, 10, and 15 nucleotides in length were incubated at a final concentration of 1 μM with 50 nM of purified $NrnB_{Bt}$ and 5 mM manganese. The reactions also contained a trace amount of 5'-^{32}P-radiolabeled RNA. Samples were removed at time intervals and analyzed by urea-denaturing 20% PAGE. (B, D) Quantification of the normalized radioactive intensity of the initial substrate depletion over time plotted as the average and SD of 3 independent experiments in (A, C).
